# Navigating the choices of decision-making in cesarean sections: medical and personal perspectives from a qualitative study in Iraq

**DOI:** 10.11604/pamj.2025.50.65.43936

**Published:** 2025-03-03

**Authors:** Maysaloon Adnan Abdul Razzak, Zainab Abdulameer Abdulrasol, Ali Fadhil Obaid

**Affiliations:** 1 Gynecology and Obstetrics, Faculty of Medicine, University of Karbala, Karbala Province, Iraq; 2 Maternal and Newborn Nursing, Faculty of Nursing, University of Babylon, Babylon Province, Iraq; 3 Pediatric Nursing, Faculty of Nursing, University of Babylon, Babylon Province, Iraq

**Keywords:** Decision-making, cesarean section, medical factors, maternal autonomy, non-medical factors

## Abstract

**Introduction:**

cesarean section (CS) rates have risen globally, prompting debates on their necessity and outcomes. This study explores factors affecting decision-making for planned CS in uncomplicated pregnancies, considering both medical and non-medical factors.

**Methods:**

a descriptive qualitative study design was employed from the period June 2022 to December 2022, comprising one-on-one conversations with Karbala midwives and obstetricians. Data were analyzed thematically to uncover factors influencing CS decisions. Descriptive and inferential statistical analysis was followed.

**Results:**

the study included 131 pregnant women, aged 20-35 (71.0%) with different educational levels. Prenatal care engagement was high, as 98.5% received care. Planned CS constituted 52.7%, and 47.3% were emergency CS. Decision factors included personal desire (22.9%), perception of efficiency and care (11.5%), and husband's desire (3.8%). Medical reasons (maternofetal factors) outweighed non-medical reasons (60.5% vs. 15.3%), with notable factors including cervical stiffness (21.4%) and fetal position/weight (17.6%). **Conclusion:** decision-making for CS involves a complex interaction of medical and non-medical causes. Healthcare professionals should understand the reasons behind planned CS to make well-informed decisions that align with each patient's unique situation.

## Introduction

Over the past few years, there has been a substantial rise in the rates of cesarean sections (CSs), often with no clear maternal or neonatal rationale [1]. The assisted reproductive tech. (ART) and elective CS are interrelated. Studies have verified that ART singleton gestations have increased odds of being delivered by CS than spontaneous gestations [2-5]. The principal focus of the healthcare regimen is assumed to be maternal healthcare [6,7]. Since there were no clear reasons for increasing rates of cesarean deliveries, the concept that some CSs were unnecessary, and no clarification for the constant increase in elective CSs, the approach of birth and associated influences must be closely assessed [8].

There are now more women making decisions about their maternity care due to the movement that supports patient involvement in healthcare decision-making. The current controversy surrounding pregnant women's wishes to have a CS has developed from discussions about topics like antenatal screening testing and the use of analgesics during labor [9].

Until recently, women who were deemed to be at low risk for pregnancy were expected to give birth vaginally. Only when possible difficulties are discovered during labor or are suspected in the prenatal period do obstetricians conduct a CS. It has been asserted in recent years that women are independently choosing the mode of birth, either out of personal preference or by the concept of “informed consent” [10]. It's becoming contentious to advocate for women with low-risk pregnancies to have decision-making authority as a mode of birth. The question of whether it is appropriate to request a CS in cases without medical indications—or, as it has come to be known" a CS per maternal request"—has been the main topic of discussion recently [11,12].

Several research studies have been carried out to investigate the perspectives of different participants, such as obstetricians and midwives, pregnant women, and the general public, regarding women's involvement in choosing a CS as the method of delivery in low-complication pregnancies [13,14]. Professionals and ethicists have disagreed over the concept of maternal autonomy in selecting a planned childbirth, and national policies differ in how these requests should be handled [15,16]. The present survey aimed to deliver sample knowledge and insight by evaluating the factors that affect the decision-making to perform CS.

**Objectives of the present study:** this study explores factors affecting decision-making for planned CS in uncomplicated pregnancies, considering both medical and non-medical factors. The specific questions we aimed to answer include: 1) what medical and non-medical factors influence the decision-making process for planned cesarean sections? 2) how do healthcare professionals perceive their roles in this decision-making process?

## Methods

**Study design:** this study employs a mixed-methods approach, integrating both qualitative and quantitative aspects to explore factors affecting decision-making for planned cesarean sections (CS) in uncomplicated pregnancies. This designation reflects the comprehensive nature of our research, which includes a thematic analysis of qualitative interviews with healthcare professionals and a descriptive statistical analysis of quantitative data collected from participants. The current study uses a descriptive qualitative design to explore factors affecting decision-making for planned CS in uncomplicated pregnancies, considering both medical and non-medical factors.

**Study setting and population:** the present study conducted in-depth one-on-one interviews with midwives and obstetricians who were engaged in the decision-making process about CS in the chosen study sites. This method was selected to explain and investigate the factors influencing decision-making for CS. Two hospitals in the province of Karbala for maternity and children were involved. Women from both urban and rural locations, with varying levels of obstetric risk, made up the population in these settings. The study sample consisted of midwives, physicians (consultant obstetricians and senior registrars) who were engaged in the decision-making process for CS, as well as labor ward midwives with varying degrees of expertise. The clinicians were purposefully selected based on these characteristics. The study excluded doctors who worked as senior house officers or who were not involved in clinical decision-making for CS at the time of data collection, as well as midwives who were not practicing in the labor ward at the time of data collection.

**Variables:** they included the sample demographical data as age, educational attainment, occupational status, type of family, presence of chronic illness, (in case of ‘yes´ what the type of illness), and habits such as smoking, alcohol and following a healthy diet. The antenatal prenatal care included a prenatal visit, prenatal checkup, and taking tetanus vaccine or not. The second part is characteristics of CS among the included pregnant women, which included type of CS, place of delivery, CS decision, type of anesthesia, complications, and why made this choice. Medical reasons for CS included stiffness of the cervix, fetal position or fetal weight, cephalopelvic disproportion, problems with the descent of the placenta before the fetus, gestational hypertension, pre-hypertension, twins, infection, placental abruption/previa, and descent of the umbilical cord. The non-medical factors for CS included preterm labor, post-term labor, fear of side effects from vaginal delivery, giving birth at a specific date, age not allowing natural birth, and failure of vaginal delivery.

### Data resource and measurement

**Data collection tool:** utilizing a single interview guide that was adopted and developed from existing literature. This guide was applied uniformly for both midwives and obstetricians, focusing on decision-making. A study tool was created through an interview guide by using open-ended questions to enable a thorough examination of the perspectives of the clinicians. Several facets of the decision-making process were covered by the questions, including the functions of obstetricians and midwives, variables affecting the selection of CS, and the interaction between medical indications and individual preferences. In order to maintain consistency and enable comparison of answers between midwives and obstetricians, the questionnaire sought to provide a comprehensive picture of the physicians' decision-making process.

**Data collection:** in Iraq, public healthcare is freely available to every resident woman, with a dedicated team of health providers and obstetricians. The counselor obstetrician assumes decision-making responsibilities regarding the privately attended cases. Midwives play a vital role in promoting normality and enhancing the childbirth experience. Their responsibilities include decision-making for low-risk cases, seeking obstetrician review when needed, and collaborating with the obstetrician to ensure maternal and neonatal safety. Despite the active participation of midwives in medium- and high-risk profile decision-making, the ultimate decision authority rests with the obstetrician. The study was conducted during the period of June 2022 to December 2022.

**Sample size:** the study included 131 pregnant women, chosen through a purposive non-probability sampling procedure to ensure participants had relevant experience concerning the decision-making process for cesarean sections. Data saturation: the notion of data saturation was the main technique used to calculate the ultimate sample size. When more interviews stop revealing fresh details or themes, data saturation sets in. Continuous data analysis was conducted in tandem with data gathering during the research procedure. Data gathering ended after interviews yielded no more novel insights or notable differences in the topics. Iterative process: the need for more participants was continually evaluated as interviews were done and analyzed. More participants were enlisted to go deeper into these areas if preliminary interviews revealed a variety of unexpected or diverse factors. A total of 131 obstetricians, midwives, and pregnant women participated in the study.

**Data analyses:** data were analyzed using a thematic analysis approach for qualitative data obtained from interviews with healthcare professionals. Quantitative data analysis: descriptive statistics were employed to summarize demographic characteristics and decision factors, allowing for a comprehensive understanding of trends within the sample population. This detailed explanation clarifies how we analyzed our data to address each study objective and research question.

**Ethical consideration:** following research ethical board approval from the health directorate (Ref: 16-2022, 112-B), the three study settings were presented to all qualified midwives and obstetric teams. The study information, purpose, procedures, and participants' rights to decline or withdraw were explained to the participants. Before each interview, clinicians had the prospect to seek clarification, the drive of the study was reiterated, and written consent was obtained before the interviews. The authenticity and transparency of the respondents and the mothers were ensured.

## Results

The results are organized according to our research questions to provide clarity and facilitate understanding of the factors influencing decision-making for planned cesarean sections (CS).

**Socio-demographic characteristics of participants:** the majority of participants were aged between 20 and 35 years (71.0%). The socio-demographic characteristics of the participants are summarized in Table 1. High engagement in prenatal care was noted, with 98.5% of participants receiving regular check-ups throughout their pregnancies. The educational background varied among participants, with a significant proportion having completed secondary education or higher.

**Table 1:** comprehensive overview of socio-demographic characteristics of pregnant women participating in the study on decision-making factors for planned cesarean sections in Karbala, Iraq

Items	Interval	Frequency	Percentage
Age	Less than 20	12	9.2
	20-35 year	93	71.0
	More than 35	26	19.8
Educational attainment	Uneducated	12	9.2
	Under graduated	52	39.7
	Bachelor	58	44.3
	Master	5	3.8
	Ph.D.	4	3.1
Professional status	Housewife	68	51.9
	Worker	63	48.1
Type of housing	With husband family	46	35.1
	Rented house	9	6.9
	Own house	76	58.0
Chronic illness	Yes	29	22.1
	No	102	77.9
Type of illness	Diabetes (11) 8.4%	Hypertension (13) 9.9%	Others (5) 3.8%
Habits	Smoking	3	2.3
	Drink alcohol	2	1.5
	Eat a healthy diet	93	71.0

**Factors influencing decision-making for planned cesarean sections:** the decision-making process for planned CS was influenced by a combination of medical and non-medical factors. Medical reasons: planned cesarean sections constituted 52.7% of cases, while emergency cesarean sections accounted for 47.3%. Medical reasons (maternofetal factors) significantly outweighed non-medical reasons, with a distribution of 60.5% versus 15.3%, respectively. Notable medical factors influencing the decision included cervical stiffness: 21.4%, and fetal position/weight: 17.6%; these findings are presented in Table 2.

**Table 2:** detailed analysis of medical and non-medical factors influencing decision-making for planned cesarean sections among pregnant women in Karbala, Iraq

Items	Interval	Frequency	Percent
Prenatal care	Yes	129	98.5
	No	2	1.5
Prenatal visit	1-2	8	6.1
	3-4	30	22.9
	5-6	52	39.7
	More than 6 time	41	31.3
Prenatal checkup	Blood pressure	129	98.5
	Urine exam	127	96.9
	Blood test	128	97.7
Tetanus vaccine	Unvaccinated	17	13.0
	1 shot	22	16.8
	2 shots	82	62.6
	More than 2	10	7.6

**Figure 1:**
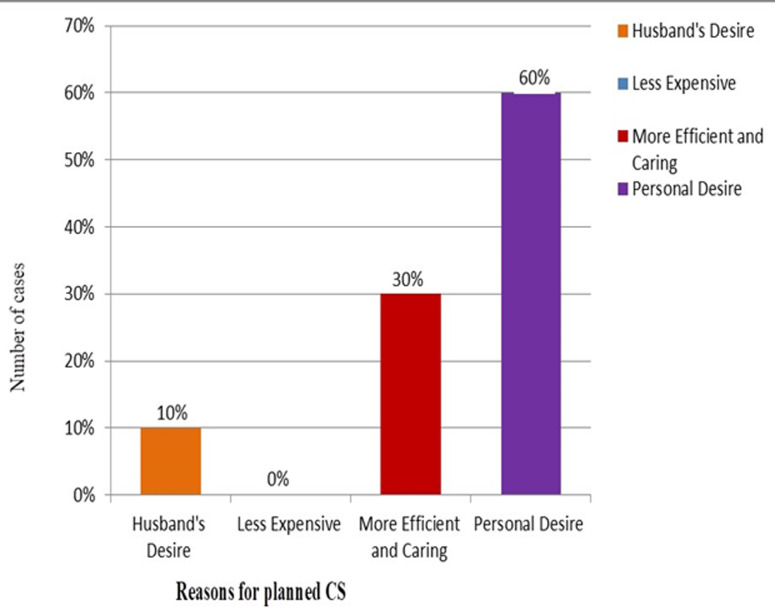
distribution of factors influencing the decision-making process for planned cesarean sections among pregnant women in Karbala, Iraq: a comparative analysis of medical and non-medical reasons

**Figure 2:**
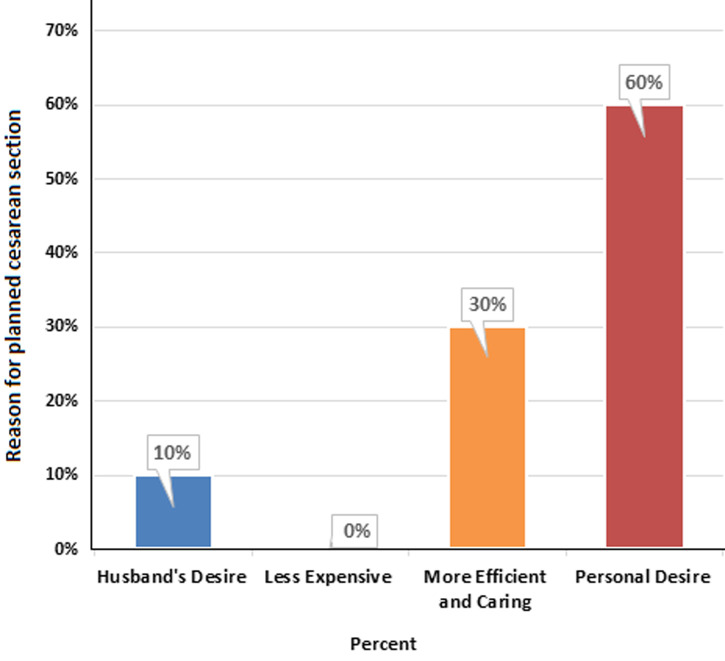
comprehensive analysis of non-medical reasons influencing the decision-making process for planned cesarean sections among pregnant women in Karbala, Iraq: insights into personal preferences and social influences

**Non-medical reasons:** the study also identified several non-medical factors that influenced women's decisions regarding planned CS: personal desire for CS: 22.9%, perception of efficiency and quality of care: 11.5%, and influence from partners (husband's desire): 3.8%. These non-medical influences highlight the role of personal preferences and social dynamics in the decision-making process, as shown in Table 3. Figure 1 depicts the various reasons influencing planned CS decisions among participants, while Figure 2 illustrates the distribution of non-medical factors impacting these choices.

**Table 3:** comprehensive overview of non-medical factors influencing decision-making for planned cesarean sections among pregnant women in Karbala, Iraq

Variables	Options	Frequency	Percent
Type of cesarean section	Planed CS	69	52.7
	Emergency CS	62	47.3
Place of delivery	General Hospital	68	51.9
	Private Hospital	63	48.1
Cesarean section decision	Before the onset of labor pain	76	58.0
	After the onset of labor pain	55	42.0
Type of anesthesia	General	59	45.0
	Spinal	72	55.0
Complications	During cesarean section	3	2.3
	After cesarean section	4	3.1
**Why make this choice**			
More efficient and caring	Yes	15	11.5
	No	116	88.5
Less expensive	Yes	0	0
	No	131	100.0
Personal desire	Yes	30	22.9
	No	101	77.1
Husband's desire	Yes	5	3.8
	No	126	96.2

CS: cesarean section

The medical measures itemized in Table 4 are significant factors for indicating a CS. Conditions including cervical stiffness, fetal position or weight, cephalopelvic disproportion, placental descent concerns, gestational hypertension, numerous pregnancies, infection, and umbilical cord disorders are among those for which it offers insights. Healthcare providers must have a thorough understanding of these medical considerations to choose the best delivery method and handle any difficulties.

**Table 4:** in-depth analysis of medical reasons for planned cesarean sections among pregnant women in Karbala, Iraq: a breakdown of factors influencing clinical decision-making

Causes	Frequency	Percent
Stiffness of the cervix	28	21.4%
Fetal position or fetal weight	23	17.6%
Cephalopelvic disproportion	22	16.8%
Problems with the descent of the placenta before the fetus	7	5.3%
Gestational hypertension, pre-hypertension	11	8.4%
Twins	9	6.9%
Infection	6	4.6%
Placental abruption/previa	6	4.6%
Descent of the umbilical cord	1	0.8%

The non-medical aspects and individual preferences that affect the selection of a CS are highlighted in Table 5. It takes into account factors including preterm and post-term labor, anxiety about the negative effects of vaginal delivery, the desire to give birth on a certain day, age-related constraints, and previous failures at vaginal delivery. Healthcare practitioners must acknowledge those non-medical elements to provide comprehensive treatment that aligns with the desires and circumstances of pregnant individuals.

**Table 5:** summary of decision-making roles and influential factors in planned cesarean sections as perceived by midwives and obstetricians in Karbala, Iraq

Causes	Frequency	Percent
Preterm labor	8	6.1%
Post-term labor	6	4.6%
Fear of side effects from vaginal delivery	13	9.9%
Giving birth at a specific date	3	2.3%
Age does not allow natural birth	2	1.5%
Failure of vaginal delivery	8	6.1%

## Discussion

In this section, we summarize the key results of our study while emphasizing its unique contributions to the field of decision-making for planned cesarean sections (CS).

**Key contributions:** our study identified specific medical and non-medical factors that significantly influence the decision-making process for planned CS among pregnant women in Iraq. Notably, medical reasons such as cervical stiffness and fetal position/weight emerged as primary considerations, highlighting the importance of clinical assessments in guiding decisions. Additionally, non-medical factors, including personal desire for CS and perceptions regarding care quality, underscore the impact of individual preferences and social dynamics on obstetric decisions. This finding suggests that healthcare providers must consider both clinical and personal factors when advising patients on delivery methods.

Furthermore, our research provides valuable insights into the roles of midwives and obstetricians in the decision-making process, illustrating how their interactions shape patient outcomes. This contribution is particularly relevant in contexts where maternal autonomy is increasingly recognized as vital to patient-centered care. Table 1 offers a thorough overview of the health and demographic details of the pregnant women who were polled, illuminating variables that can have a major impact on the well-being of mothers. Healthcare providers and legislators may find these findings useful in customizing therapies and assistance for this particular group.

In this study, the majority of the involved females are aged between 20 and 35 years. Other revisions have reported that maternal age is a significant element that influences the decision of a CS. The risk of CS rises with the age of the mothers. In a study of low-risk primiparous women, the frequency of CS rose from 14.0% in mothers below 20 years of age to 39.9% in females aged 35-39 years and more than tripled for women aged 40 years or above [17].

Around half of the females in this study had higher educational attainment. Similarly, higher education levels among women have been related to a higher rate of elective repeat CS. A survey found that females with a university degree had an increased rate of planned CS in comparison to mothers having a high school diploma. Likewise, another study showed that a rise in maternal education attainment is associated with a higher likelihood of CS [18]. Nevertheless, it's crucial to note that the influence of education on CS frequencies can differ among different racial/ethnic groups. Several studies have examined the association between women's professional status and the likelihood of CS delivery. Consistent with the findings in the current study is a German study that found that the CS rate increased for all groups over four years, but it did not specifically focus on the association with professional status [19]. However, an analysis of the “National Family Health Surveys” in India suggested a disparity in CS rates between public and private providers, and this gap was larger among females with inferior education and from poorer families [20]. Another study found that a higher education level is associated with a higher rate of elective CS [21].

Unlike other reports, the incidence of chronic illnesses was relatively low. A study based on the SNiP study found that females with chronic diseases had an increased frequency of CS [22]. The evidence suggests that maternal chronic illness or comorbidities are associated with a higher frequency of CSs and that children delivered by CS have an increased risk of developing chronic immune disorders. Healthcare providers should carefully consider the risks and advantages of CS delivery, particularly in the context of maternal chronic illness, and discuss the potential long-term implications with expectant parents.

A positive note is the high percentage (71.0%) of pregnant women following a healthy diet. The prevalence of CS can be influenced by maternal dietary patterns and nutrition levels. Ensuring a healthy diet during pregnancy can help reduce the risk of CS and promote better postpartum health for both the mother and the baby [23].

Prenatal care practices can play a role in the incidence of CS. As stated by enhanced recovery rules, antenatal care should involve educating women and their husbands about the likelihood of CS, and it is important to advise the females about what to anticipate before, during, and after the surgery [24]. The data from Table 2 underscores the positive trend of widespread engagement in prenatal care practices among the surveyed pregnant women. The comprehensive nature of prenatal checkups and the considerable coverage of tetanus vaccines indicate a proactive approach to ensuring the well-being of both mothers and their unborn children. These results may be used to evaluate the efficacy of current prenatal care initiatives and to direct future advancements in maternal healthcare programs.

The findings can be useful in improving the understanding of CS practices and influencing improvements in maternal healthcare delivery, as the comparatively low frequency of problems is encouraging. Consistent with studies, invasive placentae and ruptured uterus were two long-term impacts of CS that may raise the likelihood of complications in succeeding gestations [25]. Long-term sequels of elective cesarean have also been recognized to comprise small to moderately elevated risks of miscarriage and postpartum hemorrhage [26]. Nevertheless, several conditions, including family support, the awareness of neonatal risk, trust in God for a safe birth, and previous experience with a cesarean, may influence the decision for CS [27]. Additionally, inadequate information and an absence of combined decision-making may cause unnecessary CSs, longer hospitalizations, and increased risk of postoperative complications [28]. To encourage perfect decision-making and proper management, healthcare providers should arm women with detailed data concerning the probable risks and complications related to CS, both antenatal and postnatal.

It is important to draw attention to the fact that other thoughts, such as the female's demand and her partner's wish, the apparent risk to the neonatal life, and the provision and reassurance from family followers, can influence the decision for elective CS [27]. The decision-making might also be predisposed by the probable advantages of CS, like ease, no-labor pain, and a reduced risk of stress incontinence and uterine prolapse [25]. Furthermore, the decision for an elective CS can be affected by the type of health care provider and the variation in CS frequencies between private and public health providers, particularly for females who have less income or are less acknowledged [20].

The dual sorting revealed in Table 4 and Table 5 presents an inclusive consideration of the CSs etiologies, including both medical and personal factors. Planned CS had additional medical reasons (113/131) than the non-medical group (40/131). This highlights how problematic it may be to make decisions through labor if personal favorites collide with medical desires. Surveys revealed that planned CSs were mutually executed on medical and non-medical bases.

Several associated personal, medical, emotional, and financial factors have been considered while determining whether to have a planned CS. A clear and authentic message is frequently required between the woman, her spouse, her household, and the obstetrical team. Medical staff must furnish women with inclusive information about the benefits and risks of CS versus vaginal birth, together with non-medical and medical issues. A conversation about the detailed indicators for a CS, the likely impacts on the mother´s health and future gestations, and the emotional and psychological elements of the option may all be involved in this. The woman's aptitude to make a knowledgeable decision that is in line with her exclusive condition and medical requirements is the final aim of this argument.

**Clinical implication:** healthcare providers should prioritize open communication, ensuring pregnant individuals are well-informed about the benefits and risks of CS. Tailored support and interventions can address demographic and health characteristics influencing maternal well-being.

## Conclusion

This study illuminates the multifaceted situation of CS decision-making in Iraqi circumstances. The results highlight the complex relationships underlying medical and non-medical factors, highlighting the importance of personal preferences, care and efficiency views, and spouse influence. Proactive attention to maternal health is shown by high prenatal care engagement. The frequency of medical conditions, such as fetal position and cervical stiffness, that impact CS decisions highlight the complexity of obstetric considerations. Healthcare providers should prioritize open communication, ensuring pregnant individuals are well-informed for informed decision-making. These insights contribute to a holistic understanding, guiding tailored interventions for optimal maternal care.

### What is known about this topic

- Cesarean section (CS) rates have increased internationally, prompting deliberations on their necessity and consequences;
- Till this moment, mothers who were deemed to be at low risk for pregnancy were expected to give birth vaginally; only when possible, difficulties are discovered during labor or are suspected in the prenatal period do obstetricians conduct a CS.

### What this study adds

- This study identifies specific medical and non-medical factors that significantly influence decision-making for planned cesarean sections;
- It also highlights the importance of personal preferences in the decision-making process, revealing that many women express a desire for cesarean sections based on individual circumstances;
- It provides insights into how midwives and obstetricians interact with patients, shaping outcomes in the decision-making process.
